# Myocardial iron assessment by T1 cardiovascular magnetic resonance at 3 Tesla

**DOI:** 10.1186/1532-429X-16-S1-P309

**Published:** 2014-01-16

**Authors:** Mohammed H Alam, Taigang He, Gillian C Smith, Arun J Baksi, Ricardo Wage, Peter Drivas, Yanqiu Feng, David Firmin, Dudley J Pennell

**Affiliations:** 1 NIHR Cardiovascular Biomedical Research Unit, Royal Brompton Hospital, London, UK; 2 Imperial College London, London, UK; 3 School of Biomedical Engineering, Southern Medical University, Guangzhou, China

## Background

Myocardial T2* relaxometry at 1.5T provides reliable non-invasive assessment of cardiac iron burden and is commonly used for the diagnosis and monitoring of patients at risk. 3T CMR offers some advantages over 1.5T but iron assessment has not been routinely performed at 3T due to technical concerns regarding artefacts and rapid signal loss, as well as a lack of tissue calibration. In vitro data has shown that like T2*, T1 shortens with increasing tissue iron. At 1.5T, T1 correlates reasonably with T2* in cases of iron overload ( < 20 ms). We compared myocardial T1 measurement at 3T with measurements made at 1.5T of T1 and the established black blood (BB) T2* technique.

## Methods

A total of 73 subjects (42 male, aged 14 to 81 years) were recruited, comprising 20 healthy volunteers (controls) and 53 patients (thalassemia major 22, sickle cell disease 9, hereditary hemochromatosis 9, other iron overload conditions 13) referred for routine iron assessment. The same mid-ventricular short axis cardiac slice was used to acquire both BB T2* images and to generate T1 MOLLI maps for each subject at 1.5T (Avanto, Siemens, Erlangen, Germany), and then at 3T (Skyra). 20 subjects underwent repeat studies on the same day to evaluate reproducibility. Septal regions of interest were analyzed using CMRtools.

## Results

**Figure 1:**
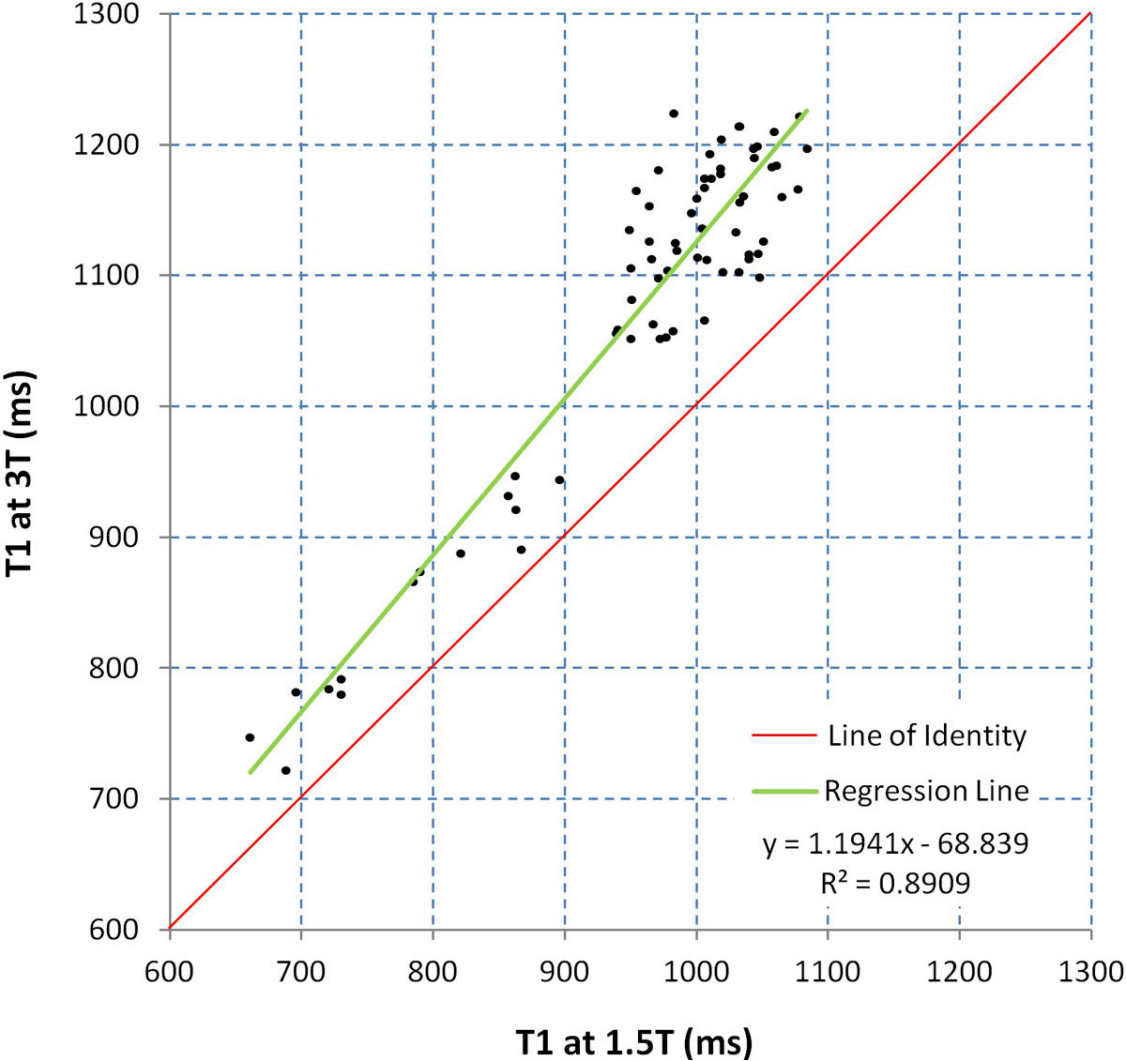
**Scatter plot showing relationship between T1 measured at 3T and 1.5T**.

**Figure 2:**
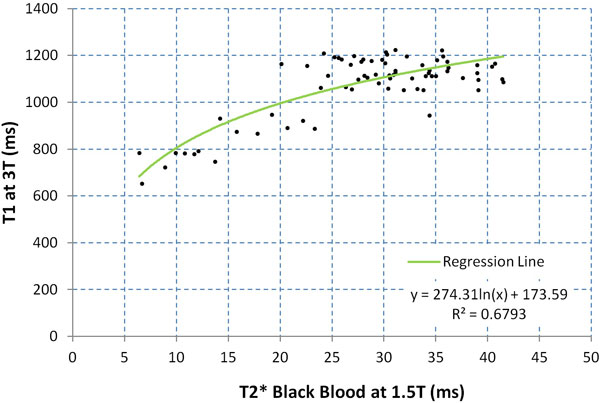
Scatter plot showing relationship between T1 at 3T and T2* at 1.5T.

There was a strong linear correlation between T1 values at 3.0T and 1.5T (R^2 ^= 0.891, gradient 1.19, intercept -68.8; Figure 1). There was reasonable correlation between T1 values at 3.0T and BB T2* at 1.5T, best described by a logarithmic relation (R^2 ^= 0.679; Figure 2). In the control group, all of whom had T2* values > 20 ms, mean T1 at 3T was 1154 ± 102 ms, thus defining the lower limit of normal as 1052 ms. All patients with T2* < 20 ms (n = 12) had a low T1 value (mean 805 ± 168 ms). Of the patients with T2* > 20 ms (n = 41), T1 was normal in 90% (n = 37). 4 patients with thalassemia with T2* at 1.5T in the clinically non-significant range (20.7-34.4 ms) had T1 values below normal at 3T (888-944 ms). There was excellent intra-observer, inter-observer and inter-study reproducibility of T1 measurement at 3T with coefficients of variation (CoV) of 0.84%, 0.89% and 1.45%.

## Conclusions

In this analysis, T1 mapping at 3T using a MOLLI sequence identified all individuals with significant iron loading as defined by the current gold standard T2* technique at 1.5T, and is highly reproducible. A linear relation between T1 values obtained at 3T and 1.5T was observed. There is a possible clinical role for T1 mapping at 3T if T2* at 1.5T is not available. Considerable further work on calibration and clinical validation would be necessary however, as the scatter between T1 (3T) and T2* (1.5T) results is not trivial, and would lead to uncertainty in risk assessment in individual patients.

## Funding

This research was supported by the NIHR Cardiovascular Biomedical Research Unit at Royal Brompton & Harefield NHS Foundation Trust and Imperial College London.

